# A Qualitative Study to Describe the Nature and Scope of Street Medicine Programs in the United States

**DOI:** 10.3390/ijerph21121623

**Published:** 2024-12-04

**Authors:** Teresa Medellin, Leticia R. Moczygemba, Whitney Thurman

**Affiliations:** 1 College of Pharmacy, University of Texas, Austin, TX 78712, USA; tmedellin@utexas.edu; 2 School of Nursing, University of Texas, Austin, TX 78712, USA; wthurman@utexas.edu

**Keywords:** street medicine, homeless healthcare, harm reduction

## Abstract

Street medicine is a health delivery model designed to provide direct patient care to people experiencing unsheltered homelessness where they are physically located, whether that be on the streets or in encampments. The model has developed in response to the barriers people experiencing homelessness (PEH) encounter when accessing care through traditional points of access such as primary care clinics. Street medicine programs are rapidly emerging across the United States (U.S.) in response to the health needs and challenges associated with care access and coordination for unsheltered homeless individuals. Although street medicine is a rapidly growing field, existing street medicine programs have rarely been studied collectively, limiting our understanding of the nature, scope, and range of street medicine programs in the U.S. This study examined 13 programs from across the U.S. to develop a broad characterization of street medicine programs. Results from interviews with representatives from each of the 13 programs show that there is a high degree of variability among the structure, operations, and scope of care of street medicine programs. However, consistent among street medicine programs is the adoption of a patient-centered approach to care and the use of harm-reduction principles. Street medicine programs are also highly engaged with community partners and affiliate organizations that work in their local and regional areas. Because street medicine programs often serve as a bridge between formal healthcare entities and PEH, street medicine offers a strategy for reconnecting individuals to vital healthcare services.

## 1. Introduction

In 2023 on a given night in the United States (U.S.), approximately 653,100 people experienced homelessness [1]. Among the homeless population, 40% are unsheltered, meaning that their primary nighttime residence is a place not suitable for habitation (e.g., sidewalks, bus stations, or abandoned buildings). The unsheltered homeless population in the U.S has complex health and social needs and, in general, has a higher burden of illness compared to the sheltered homeless population. Lack of consistent access to running water, nutritious food, and spaces protected from the elements increases the risk of unintentional injury (e.g., burn, frostbite, and falls), chronic and acute illness, as well as premature death [2]. A study performed in Boston, Massachusetts found that unsheltered homeless persons have an all-cause mortality rate approximately 10 times higher than that of the general population of Massachusetts [3]. The same study found that the all-cause mortality rate for unsheltered homeless individuals is approximately three times higher than that of the sheltered homeless population. In comparison to sheltered homeless, which refers to individuals living in homeless shelters or transitional housing, unsheltered individuals are more than four times as likely to report a physical health condition such as chronic lung or heart issues (84% vs. 19%), nearly one-and-a-half times as likely to report a mental health condition (78% vs. 50%), and more than five times as likely to report a substance use condition (75% vs. 13%) [4]. Additionally, compared to sheltered homeless individuals, unsheltered individuals are 25 times more likely to report experiencing all three conditions concurrently (50% vs. 2%) [4].

Despite the complex health needs and high disease burden of the unsheltered homeless population, there are many barriers that prevent this population from accessing both primary and acute care. Inconsistent access to a phone and transportation, lack of legal identification such as a driver’s license, concerns about cost, mental illness, and substance use make it almost impossible to access care and maintain continuity of care [5]. In addition to logistical and structural barriers, mistrust of healthcare workers, discomfort in traditional healthcare settings, and past experiences of trauma in healthcare settings discourage many unsheltered individuals from seeking healthcare [6]. This combination of structural barriers and personal factors makes it difficult for unsheltered homeless individuals to access clinical services. Because unsheltered individuals have high rates of morbidity but low access to care, they are at a high risk for using emergency department services, and they are more likely to receive acute care rather than preventative care [2,7]. Chronically homeless individuals make up approximately 33% of all emergency department visits. Of these visits, up to 80% could have been addressed via preventative care [8].

The unmet health needs of the unsheltered homeless population and the barriers this group faces in accessing traditional healthcare services prompted a move to bring healthcare to the unsheltered population where they are physically located. In 1992, one of the first programs to provide these services emerged in Pittsburgh, Pennsylvania. Healthcare models that seek to provide direct care to patients experiencing homelessness in their own environment (e.g., on the streets or in encampments) can broadly be classified as street medicine [9]. The Street Medicine Institute, the global leader in the field of street medicine, describes the general aim of street medicine as providing healthcare to patients on their own terms and in their own environments to reduce barriers to care [9]. Although street medicine is an evolving field, with an estimated 150 programs across the U.S., existing street medicine programs have rarely been studied [10]. Thus, the aim of this qualitative study was to learn more about the operational aspects of street medicine programs in the U.S. The specific objectives were to (1) understand qualitatively the nature and scope of urban street medicine programs in the United States and (2) explore how street medicine programs operate in relation to local health infrastructures.

## 2. Materials and Methods

Semi-structured interviews were conducted to answer the study objectives. Potential participants were identified from the attendee list of the 2021 and 2022 Street Medicine Institute Symposium and contact pages from websites of active street medicine programs in the U.S. Eligibility criteria included team members from street medicine programs in the U.S. who met the following criteria: (1) Operated multidisciplinary teams that included at least one licensed independent medical practitioner; (2) Provided basic medical care; (3) Provided services to communities at least once per month. Emails requesting a virtual interview were sent out to prospective participants. The email invitation outlined the study goals and the interview process. All 13 programs that were sent an interview request agreed to participate in the study.

### 2.1. Interview Guide

A semi-structured interview guide was developed based on the current literature related to street medicine and healthcare for homeless populations [11,12,13,14]. The interview guide included 14 questions. The first question asked the participant to describe their role in the street medicine program and how long they had been in this role. Three of the interview questions related to the philosophy and values of the street medicine program. Four questions were related to program operations and logistics; these questions asked about the services provided, the programs’ resources and funding, how the team identifies communities to work with, and the frequency with which the street medicine team returns to sites. Three questions asked about the development of care plans and outcome measurement. The remaining three questions asked about program facilitators and challenges. See Appendix A for the interview guide. The duration of interviews ranged from 45 min to one hour.

### 2.2. Data Collection and Analysis

The semi-structured interviews were conducted virtually over Zoom from March to April 2022. All participants provided verbal consent allowing interviews to be recorded and the Zoom transcribing function to be used. The transcripts produced by Zoom were then checked and revised, when needed, for accuracy. Responses from interviews were analyzed using line-by-line open coding to group similar concepts into themes and categories for each objective. After inductive coding, the literature was used, when applicable, to finalize code names. A codebook was used to document codes. The codebook was updated throughout data analysis as needed. Initial coding was predominantly conducted by one researcher and reviewed by two other researchers. Discrepancies were discussed by the group until consensus was reached, usually using the literature to guide the final terminology of themes and categories.

## 3. Results

Team members from 13 street medicine programs from 10 states across the United States were interviewed. Programs from the following states are included in this study: California, Texas, Pennsylvania, Massachusetts, Illinois, Colorado, Florida, Washington, Oregon, and Rhode Island. All participants had been working with the street medicine program for at least one year. The participants included physicians (*n* = 3), physician assistants (*n* = 2), nurses (*n* = 2), community health workers (*n* = 2), medical students (*n* = 2), a social worker (*n* = 1), and a director of services (*n* = 1). Both medical students held leadership positions and had longitudinal engagement with the program. After qualitative analysis of the interview transcripts, three main themes were identified: local context, variable program components, and uniform program components. Each theme has subcategories that include features unique to the larger category. The following section details the findings from each theme and how these findings relate to the nature and scope of street medicine programs and how street medicine programs operate in relation to local health infrastructures in the U.S.

**Figure 1:**
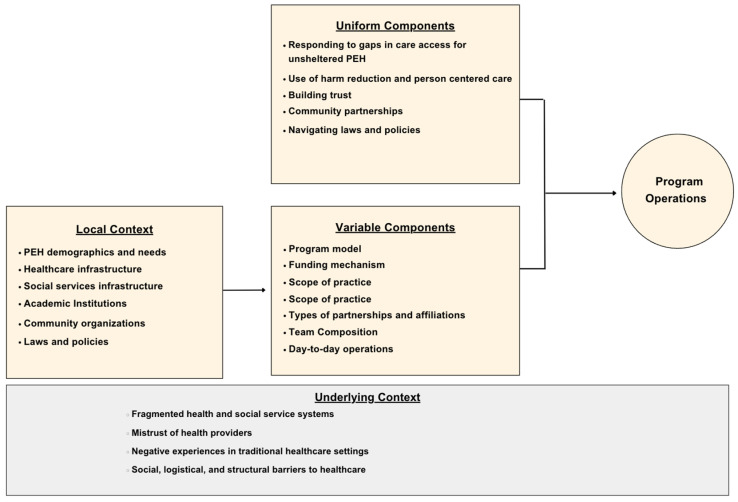
Components of street medicine programs.

Figure 1 presents a schematic of the overall findings which characterize how local context influences certain variable components of a street medicine program such as the institutional affiliations, community partners, and funding mechanisms. Despite the variation that exists among programs, there were key elements that were uniform among street medicine programs, regardless of the local context, program model, or funding mechanism.

### 3.1. Local Context

The local context describes the social, economic, political, and healthcare infrastructure in the area in which the street medicine team operates. This includes the payer mix, number and type of social service programs, the funding for homelessness response services, and the number of health clinics serving low-income individuals. State, county, and city policies and infrastructures led to significant variation in the local contexts across the different areas in which street medicine programs operated. For example, there were differences among state and local policies regarding insurance coverage (e.g., Medicaid-expansion and county medical coverage programs).

The local context also includes the demographics of the local homeless population and the resources available to people experiencing homelessness (PEH). For example, some programs operated in an area where the homeless population was a relatively small number of people (<100), while other programs serviced an area that had thousands of PEH. Interview participants articulated how city- or region-specific dynamics informed their operations such as the types of services they provided, weather considerations, and the places in which they provided services. Furthermore, opportunities available for street medicine programs to collaborate with or be sponsored by local entities such as community organizations, hospitals, and academic institutions were identified as another important aspect of local context. Critical consideration of the local context allows programs to efficiently leverage resources in the community while responding to the unique needs of the local population of PEH.

#### Navigating Laws and Policies

Respondents discussed how local efforts to minimize the visibility of homelessness such as criminalizing sleeping in public spaces and encampment clearings by city officials can significantly disrupt the operations of street medicine programs. Interviewees mentioned that following a sweep, it can be challenging for them to regain contact with patients, especially patients without phones. Sweeps also disrupt programs’ efforts to establish their presence within the community and build trust with those living in the encampment. The following quote demonstrates the challenges a street medicine team faces following a sweep:

*“[Encampment clearings] have been the hardest challenge right now because we will see a patient on Tuesday, get them started on meds, and serious meds for things like opioid use disorder. We give them a week’s worth of meds, and we come back to the camp, and it’s been cleared out. A lot of our patients don’t have phones, and a lot of our patients go by street names. So, it’s hard to follow up with our patients.”*

In addition to sweeps, other policy challenges mentioned by programs included the lack of Medicaid expansion in their state and legal document requirements (e.g., state-issued identification cards and birth certificates) to access health and social services external to their program. Additionally, program representatives broadly discussed the legal and policy requirements their programs must navigate to deliver services. These requirements included insurance billing restrictions and restrictions on the services that can be provided by the health providers.

### 3.2. Uniform Program Features

Overall, there was consistency among respondents regarding the philosophy and beliefs of their street medicine programs’ overall goals for minimizing barriers to care for PEH. Each program’s philosophy broadly reflected the philosophy of the Street Medicine Institute: Go to the people and meet them where they are. The philosophy of going to the people and meeting them where they are represents the programs’ recognition that this population’s needs for healthcare services and delivery must be addressed differently than stably housed individuals. Participants emphasized centering the patient and their experience, needs, and goals in all interactions. Participants also consistently expressed their use of a harm-reduction and trauma-informed approach to their philosophy and delivery of care.

#### 3.2.1. Responding to Gaps in Care Access for Unsheltered PEH

Each program was developed in response to the failures of traditional health systems to provide adequate healthcare to the unsheltered homeless populations in their community. Program representatives articulated the imperative to provide a better option for healthcare delivery for PEH, particularly for those that live unsheltered. Each program developed in different ways and on different timelines due to the availability of resources, the founding members’ vision for the program, and the local need. However, fundamental to the program’s establishment was the desire to improve healthcare access and health outcomes for PEH.

#### 3.2.2. Harm Reduction and Person-Centered Care

Program representatives emphasized their use of harm-reduction principles as a way of centering patients and as a necessary strategy for providing quality healthcare that is non-shaming. The principles of harm reduction allow providers on street medicine teams to devise care plans that are feasible for patients and compatible with their life and the resources available to them. A consistent theme was that programs have incorporated harm-reduction principles into their model of care. For example, programs universally expressed using non-judgmental language when engaging patients as well as educating patients about safer sex practices. Most programs either provided safer drug use kits (e.g., sterile needles, sterile supplies, and overdose reversal agents) or partnered with an organization that did so.

Participants reported that programs’ strategies for harm reduction are not limited to promoting safer substance use or safer sex practices. They see harm reduction as a way of navigating the challenges associated with being homeless. Indeed, one participant described the approach as, “You go to where the people are, and you build a relationship with them. The therapeutic relationship itself is oftentimes the biggest thing that we can offer. And then using the principles of harm reduction, using the medical knowledge and skills that we have after that, not just forcing it upon someone, but allowing them to say, ‘Hey, I do want help with this or can you get me connected to that?’”.

In addition to a harm-reduction approach, a broader emphasis on patient-centered care describes a feature of street medicine programs. A patient-centered approach focuses on the experiences, needs, values, and goals of a patient when devising care plans. As stated by one participant, “We go out without an agenda. We just keep going out, and we’ll just say ‘hey, we are with street outreach, is there anything we can help you with today?’” Programs use this approach to build trust with patients and also to provide care plans that are compatible with the patient’s reality. Participants also recognized that many PEH have had significant experiences of trauma. There was consistent acknowledgement among participants about the mistreatment of PEH that can occur within hospitals and medical clinics. Because of the prevalence of trauma among PEH, programs expressed the use of trauma-informed language and principles within their practice.

#### 3.2.3. Building Trust

Respondents described PEH’s mistrust of healthcare providers as a significant barrier to care for the unsheltered homeless population. As a result, street medicine programs have developed strategies to build trust with PEH. These strategies largely center on the consistency of services and accountability. This approach allows the street medicine team to integrate PEH needs and values when working with PEH to co-develop care plans.

Consistency was widely mentioned by respondents as essential to building trust. Consistency is how often programs are returning to sites and engaging with unsheltered populations. Respondents described how consistency and continuity in interactions between the street medicine program and patients were important because failure to return to sites disrupts the trust building process. Related to consistency, respondents discussed accountability as another strategy to build trust. Following through on agreements made with patients was mentioned in interviews as an important feature of this strategy. To maintain accountability, street medicine teams are aware of what they can realistically provide and achieve given the resources available to them: “If you say, ‘Hey, I’m gonna do something’, then you better have the resources, or at least the capacity to follow through. So, you know we’re careful about letting them know what we can provide. It has to be within our scope and within our realm”. Teams also avoid overpromising or falling through on a promise because it negatively impacts trust building and can reproduce the harm this population has experienced. The harm-reduction and patient-centered care philosophies, discussed in the previous section, also help facilitate building trust between street medicine teams and PEH. Some participants also described how their program employs outreach workers who have lived experience of homelessness. These individuals can provide guidance to the team about approaching certain scenarios, and they can also facilitate trust building with PEH by being able to relate to their experiences.

#### 3.2.4. Community Partnerships

A key theme that emerged during interviews was the role community partnerships have in supporting and informing the development and operations of street medicine programs. For street medicine programs in early phases of development, established organizations familiar with the local unsheltered homeless population can inform the team about the demographics, needs, and locations of the local unsheltered homeless population. Programs discussed that partnering with organizations already trusted by PEH can improve PEH’s willingness to engage with street medicine teams. Street medicine teams also learn from organizations that are well-established in the community and in homelessness response services. This collaboration often informs operations and interventions to ensure that their delivery of care is appropriate and attentive to the reality of unsheltered PEH. For example, one participant shared the following: “We partnered with community health paramedics who had been doing this work 10 years prior to us. They were already out in the community building trust, so they could vouch for us. So, initially we piggybacked on their success out in the community, and then overtime, we began to develop that trust”.

### 3.3. Variable Program Features

Variable program features refer to the aspects of street medicine programs that differ among programs. Based on interview findings, the local context of a street medicine program significantly shapes and informs how a street medicine program will develop their program operations. Moreover, certain variable components of a program such as the program model and institutional affiliation will influence other components such as the level of care and funding mechanisms. Key variable features of a street medicine program include the following: program model, institutional affiliations, funding mechanism, level of service, team composition, and day-to-day operations.

#### 3.3.1. Program Model

Program model refers to the general operating structure of a street medicine program. Interviews with key informants led to a broad classification of street medicine programs into the following categories: staff model, hybrid staff–volunteer model, and a volunteer model. See Table 1 for a description of these categories.

The model under which a street medicine program operates is not static. Interview participants described how early in their development, they might operate under a volunteer model but then transition to a hybrid model or a staff model as either funding and/or affiliations change. Programs expressed that remaining within one model or transitioning to another model is largely based on the leadership’s vision for the future of the program as well as the funding resources available for staffed positions. Moreover, some volunteer programs expressed no long-term plan to transition to a different model because the volunteer model was most appropriate for the context and goals of the program.

#### 3.3.2. Funding Mechanisms

The participants articulated various mechanisms and resources that they use to financially sustain their operations. There is not a uniform strategy for funding street medicine, and each program leverages multiple resources to make their operations financially viable. Based on the interviews, the source and amount of funding a program receives change over time and will vary based on many factors such as grant funding, county budget, or sponsorships by external partners. Funding sources mentioned in participant interviews included some combination of the following: grants, philanthropic donations, county funding, federal funding, insurance coverage, or funding through an affiliated partner such as a county Department of Public Health.

Funding for a street medicine program can be further classified into funding for medical goods and funding for staffed positions. Volunteer program models did not need to pay staff members, so their funds went to paying for medical equipment, medical supplies, and other resources (e.g., food, water, and hygiene products) used during street runs. Some volunteer programs, particularly student-led programs, expressed that they were able to receive some of their medical equipment and supplies through their institutional affiliate (i.e., academic institution). This reduced the amount of money the program needed to secure to sustain their operations. However, some student-led programs encounter challenges with securing faculty to supervise them on street runs: “The students have been able to get some great little mini grants to get supplies and backpacks things like that. But all the time that I spend [with street medicine] is just pro-bono. So, there’s no protected time for faculty to go”. Staff-based and hybrid programs had to secure funding or donations for supplies as well as stable funding for staff positions. Programs that relied primarily on donated supplies or philanthropic donations to support their operations expressed the challenges associated with expanding their operations. Inadequate or inconsistent funding constrained programs’ operations, particularly in terms of acquiring medical equipment or securing paid staff. Programs that had secured more consistent funding such as revenue from billing insurance or county funding had an increased capacity to expand operations and increase their scope of practice.

#### 3.3.3. Scope of Practice

**Figure 2:**
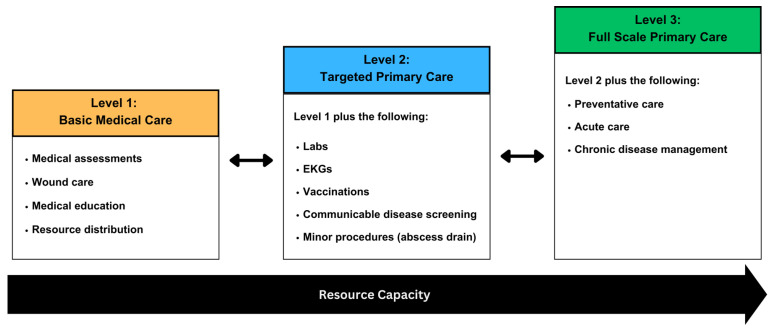
Street medicine levels of care.

In addition to program funding, the range of services offered by a program was influenced by many factors, including the medical equipment and supplies available to the program, the provider type, liability coverage, and organizational goals. Programs that were not fully dependent on volunteers for the provision of services were more likely to achieve higher levels of care. Based on the interviews, the scope of practice of street medicine programs was organized into three levels described below (see Figure 2).

The Level 1 program capacity includes basic medical assessments, basic wound care, the distribution of over-the-counter (OTC) medications, and medical education. Level 1 primarily provides information to patients about medical and social resources available in the community. In some programs, if there is an institutional affiliation or a partnership between the street medicine program and a brick-and-mortar site of care (e.g., a clinic or hospital), the street medicine team can facilitate a warm handoff for services. A warm handoff refers to a transfer or referral from one service provider to another. Special effort is made to engage all three parties (the old and new providers as well as the patient) to improve the referral process to the new provider.

In Level 1 programs, the medical services the team can provide is limited, so the ability to provide information about sites of care to patients needing medical attention is important. In addition to providing information and connecting patients to clinics, when possible, Level 1 street medicine programs play a valuable role in assessing patients’ conditions, addressing acute needs, as well as providing resources about places where the patient can receive additional care. However, these programs are limited in their ability to reduce many of the gaps associated with healthcare access because patients still must go to another site to receive primary healthcare services.

The Level 2 program capacity includes Level 1’s abilities but has additional capacity for targeted primary care. For example, on the streets, they might use some combination of electrocardiograms, lab drawing, screening for communicable disease, vaccines, and abscess draining. They might also write prescriptions for medications and dispense medications. However, these programs do not have the capacity to offer the full range of primary care services described in Level 3 due to resource limitations or the providers’ scope of practice. Thus, Level 2 programs deliver targeted primary care in addition to basic medical care. Like Level 1 programs, if there is an institutional affiliation or a partnership between the street medicine program and a site of care, the street medicine team can facilitate a warm handoff.

Level 3 represents programs that have full-scale primary care. Thus, these programs have the capacity to provide not only certain aspects of primary care, but the full range of services that are included in primary care, including preventative care, acute care, chronic disease management, minor surgical procedures, coordination of care with specialists, and options for sexual and reproductive healthcare. Preventative care can include physicals, screening tests (i.e., labs, EKGs, and STI screenings), and immunizations. Level 3 programs can similarly facilitate warm handoffs with other providers and sites of care if they have affiliations or partnerships.

Interview participants expressed how the stability of remaining in a level or moving up a level depends largely on the resource capacity of a program. Program representatives expressed in interviews how their scope of practice can change based on multiple factors including the expertise of the providers, loss/acquisition of medical equipment, changes in liability coverage, and notably, funding or affiliations. The ability to provide resources and/or facilitate a warm handoff with other health clinics or organizations was an important aspect of street medicine teams to address patient health needs that the team did not have the resources or expertise to manage.

Additionally, study participants noted that working in a street medicine context requires that they deviate from standard clinical guidelines to provide care that is compatible with the reality of being homeless. “Maybe the evidence says, you should do a seven-day course, but we know for our population that doing a shorter course has better adherence”. Participants expressed that developing care plans that are feasible for patients is essential to meeting the needs of those on the streets. However, without standardized guidelines adapted for a street medicine context, it can be a challenge to do so appropriately.

#### 3.3.4. Type of Partnerships and Institutional Affiliations

Collaboration with multiple external organizations was a uniform feature of street medicine programs and viewed as critical to being successful. As one respondent stated, “There are a lot of people doing this work. [Our street medicine program] is one piece of a larger puzzle, so we work closely with other organizations because we cannot do street medicine on our own”. Another respondent said, “We’re constantly working with outreach organizations in our county and trying to build those connections because we’re one small team for a very large county where there are lots and lots of people living outside”. However, the types of partnerships and affiliations varied from program to program. Based on interview findings, street medicine programs engage in both formal and informal partnerships with external organizations. Formal partnerships are those in which the street medicine program has a contract with an external organization to fulfill certain roles and responsibilities. Informal partnerships are those in which there is no contract between the two entities, but referrals or exchanges of resources, services, and information occur. Among the programs interviewed, formal partnerships were less common than informal partnerships, and it was common for street medicine programs to have multiple partnerships.

In addition to partnerships, some street medicine programs expressed the role that institutional affiliations play in their program. An institutional affiliation occurs when some larger institutional entity (e.g., a hospital, academic institution, public department, or health organization) sponsors a program. The program will operate in the name of or as part of the larger institutional entity. The larger institutional entity can give the street medicine program access to personnel (volunteers and/or paid staff), supplies/equipment, and partnerships. Interview participants described how institutional affiliates play an important role in establishing and sustaining the program and defining the goals, character, and structure of a program. However, the extent to which an institutional affiliate will be involved with the program is variable.

**Figure 3:**
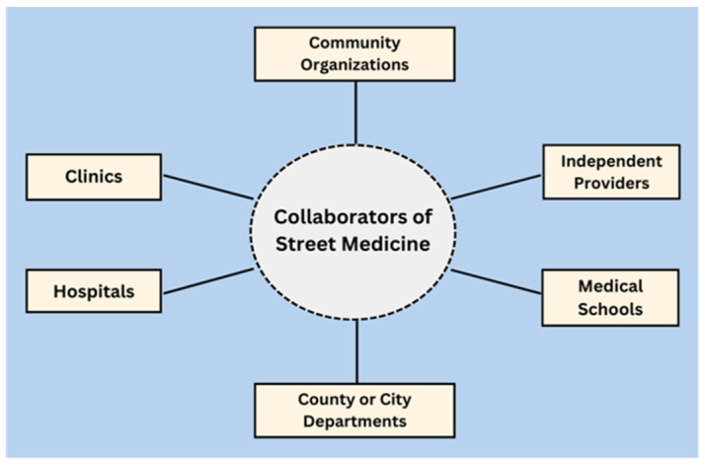
Collaborators of street medicine.

In Figure 3, the various collaborators of street medicine programs are depicted. This graphic includes the groups or individuals that create street medicine programs and establish partnerships or affiliations with the programs. These different entities interact and collaborate in various ways based on the needs of the PEH population as well as the needs, goals, and resource capacity of the involved collaborators.

Table 2 summarizes how street medicine programs in this study varied by their level of care, affiliations, and program model. Based on the findings from this study, higher levels of care are associated with a staff-based program model. Additionally, programs that are part of an FQHC are associated with higher levels of care. Volunteer-based programs that are primarily organized and facilitated by health students are more limited in the types of care services that can be provided. Staff models were also the most prevalent program model among the study participants.

#### 3.3.5. Team Composition

A street medicine program typically consists of an administrative team and a street team. The administrative team manages the financial, legal, and logistical aspects of a program; its roles usually include an executive director, a medical director, and a director of operations. The street team comprises the individuals that go out onto the street to deliver the care. Interview findings suggest that it is not uncommon for an individual on the street team to also have a position on the administrative team.

The composition and number of personnel are highly variable from program to program. However, all programs have at least one licensed independent medical practitioner. Based on interviews, the medical practitioner can be a volunteer who practices with the street medicine team outside of their normal work requirements, or the medical practitioner can be staffed by an affiliated institution or organization to work full- or part-time with the street medicine program. Street medicine programs that are affiliated with an academic institution often engage health professional students, residents, and attending faculty from the institution.

A street team may also include a caseworker, a behavioral health specialist, an outreach worker/community navigator, and other roles that manage non-medical needs of the unsheltered population. However, the inclusion of these roles depends on the resource capacity of a program as well as the needs of the patient population and the goals of the program. Some programs expressed a desire to have additional team members to address the mental health needs and social needs of those to whom they provided services. However, limited resources usually impeded these programs from acquiring individuals to fill the roles. In programs that did not have additional staff or volunteers dedicated to these non-medical roles, the existing staff usually attempted to fill the role in some way. For example, medical personnel described how they might assist a patient with completing benefits applications or navigating social services.

#### 3.3.6. Day-to-Day Operations

Each program has its own strategy for establishing the day-to-day operations of the program. These operations include identifying which areas of the city the team will provide their services in, the schedule of the street team, and their strategy for returning to the sites. Various factors such as the size of the unsheltered homeless population, the resource capacity of the program, the presence of other organizations doing similar work, and the goals of the program influence the day-to-day operations. However, consistent among the programs is that they work with organizations in the city to identify areas of great need, and they listen to people living on the street for leads on where their services might be needed. Local outreach organizations also refer individuals who have a hard time getting into clinics to street medicine teams. The documentation of encounters was also variable among programs. Each program uses some version of an electronic medical record (EMR); however, programs differ in the extent to which they use the EMR and what they do with the data collected.

## 4. Discussion

Overall, while the philosophy of street medicine was consistent across the programs interviewed, the study findings revealed a high degree of variability among programs’ scopes and operations across the U.S. This was not surprising given that fundamentally, street medicine models are inherently adaptable to be responsive to the local context impacting homeless services. In fact, the Street Medicine Institute, the forefront leader of street medicine in the U.S., offers resources intended to support program development and operations but does not prescribe a uniform organizational or operational structure for street medicine programs [9]. As a result, programs in the U.S. have taken multiple paths and engaged different actors as they build their programs.

A key consistent feature of street medicine programs expressed by study participants from each program is a commitment to harm-reduction principles. While the specific strategies differed, each program identified harm-reduction principles as essential to street medicine. These findings align with the existing literature about the wide-spread commitment to and endorsement of harm-reduction principles in street medicine [15,16,17,18]. Harm reduction has gained popularity over the years not only in the substance use and mental illness fields, but as a broader health approach to reduce the harm associated with various behaviors. The study participants emphasized that the use of harm-reduction principles is a critical feature of their process of building trust with PEH. The existing literature supports the anecdotal evidence cited by practitioners of street medicine that the implementation of harm-reduction practices improves trust and reduces stigma [15]. Not only is harm reduction viewed positively by patients, but harm-reduction interventions have also been found to be an effective approach for substance use disorder in a range of settings and populations [16].

Because street medicine emerged to address the unique needs of PEH, a strong emphasis on patient-centered care and trust building is a core component of street medicine programs [9]. The study participants noted critical ways that their delivery of care differs from mainstream health services, such as meeting people where they are, building trust, and devising health plans attentive to the realities of homelessness. The intentional incorporation of harm reduction and other trust building strategies suggests an important way that street medicine differs from traditional medicine. Additionally, patients with lived experience of homelessness are more likely to avoid care and less likely to follow through with providers’ recommendations if they lack trust or perceive stigma. So, the use of person-centered care and strategies to build trust indicates how street medicine might be successful at reengaging people who have been marginalized from healthcare [6].

In addition to harm-reduction practices, the participants identified engagement with community partners that are well-trusted by PEH as a key facilitator of program success. These community partners are generally aligned with the goals of street medicine models to employ harm-reduction approaches, serving as a foundation for collaboration. Partnerships between primary healthcare providers and community organizations can strengthen the efforts of healthcare providers, particularly when there are shared values, beliefs, and attitudes [17]. Community partners can provide street medicine programs with important context regarding the local homeless population, and they can serve as important sources of information and guidance. Furthermore, these partnerships can facilitate trust building between the street medicine program and the broader community of PEH by legitimizing the program’s work [18]. Because of the key ways that positive community partnerships can facilitate operations, programs should strongly consider partnering with organizations that are established in the community and are viewed positively by PEH.

Responding to homelessness requires a collaborative, multidisciplinary approach, so street medicine programs articulated their engagement with entities including public health agencies, hospitals, and various community-based healthcare and social service providers. Street medicine’s partnerships with other healthcare entities including traditional, mobile-health, and shelter-based clinics position street medicine across the broader healthcare system [19]. This capacity for street medicine to engage patients on the street and connect them with brick-and-mortar health clinics suggests that street medicine programs can serve as a bridge between PEH and formal healthcare systems.

As mentioned previously, collaborating with other actors involved in homelessness response is vital to maximally respond to the needs of PEH. However, not every partnership will necessarily facilitate program success. If there is misalignment among values, goals, or operating strategies, partnerships can impede the efforts of a program [20]. Despite the challenges that street medicine programs can experience with some partners, the programs rely on many of the services offered by other entities to maximally respond to the health and social needs of their patients [20]. Street medicine programs should thus optimally seek partners that are aligned with the values and goals of their program. However, it is likely that street medicine programs will have to engage key partners that have values and goals that might be misaligned with the broader goals of street medicine.

Street medicine programs that have partnerships with established organizations such as hospitals or health clinics can benefit from their expertise in financial, legal, and administrative matters. Although not investigated directly in this study, a stable funding mechanism or a financially invested institutional affiliate is necessary to facilitate program operations and program stability. Furthermore, there appeared to be an important interplay between these three features such that a program’s affiliations often impacted the robustness of the program model and the funding mechanism (and vice versa). For example, street medicine programs that are part of a federally qualified health center have access to an existing infrastructure that can facilitate specialty care or social service referrals [19]. Programs that articulated a stable or reliable funding source, such as insurance billing or county/city budget allocations, were more easily able to acquire medical resources and equipment for higher levels of care. This suggests that the most financially stable mechanisms for street medicine programs are tapping into funding strategies that exist in traditional health systems (e.g., billing for service). Although securing a well-established funding source is important to increase program capacity, the current funding options for street medicine are, for the most part, inadequate [20]. A positive step toward payment for street medicine was the Center for Medicare and Medicaid’s (CMS) inclusion of the “outreach site/street” as a place of service to indicate where care was delivered for potential reimbursement from payers for these services (effective October 2023) [21]. This recognition by CMS highlights the importance of patient-centered care (in this case, meeting people in their environment) and has potential to be adopted by other payers.

Within the street medicine community, there is informal guidance and peer program mentoring via informal networks such as conference chat groups. However, many gaps persist regarding best practices and important clinical considerations for street medicine [22]. Standard clinical guidelines are not well-adapted to the reality of unsheltered homelessness and thus create challenges for providers of street medicine. Study participants and other providers of street medicine note that they often must adapt clinical guidelines in order to practice “reality-based medicine”, meaning that their health recommendations are compatible with the reality of homelessness [19,20,23]. However, adapting standard clinical guidelines for a street medicine context is highly dependent on the expertise of providers operating out of their respective programs. As a result, practices are likely highly variable across the U.S. and can lead to variations in programs’ ability to provide high quality care.

The findings from this study echo others who have advocated for more formalized guidance about best practices relevant for street medicine. In a study based on California street medicine programs, over three-fourths of the participants wanted more training specific to street medicine [20]. The development of guidelines for street medicine operations should draw upon the experience of street medicine providers to ensure that recommendations are realistic for the context of street medicine and ensure the highest level of care for PEH. Stakeholders should identify core elements of a street medicine program and understand how they can be standardized across street medicine contexts in the U.S. This standardization is important for making street medicine more easily understandable and thus advocate its role and value to funders and others involved in the expansion of street medicine. This is especially true as payers begin to consider reimbursing providers for these services. Advocating to the larger system about street medicine’s role can increase the options available for street medicine programs to develop well-funded and sustainable operations. This study offers a broad characterization of street medicine programs that can inform programs and the broader public about the various components of active street medicine programs. The work also provides an overview of the different actors and entities that are connected to street medicine which can guide emerging street medicine programs as they develop their program.

Limitations: This study involved a limited number of participants, which may restrict the generalizability of the results to a broader population. The findings may not reflect the experiences and perspectives of individuals outside of the sample group.

## 5. Conclusions

Street medicine programs address the health needs of PEH by creating low-barrier pathways to health services. The street medicine care model is flexible to be responsive to local contexts and available healthcare infrastructures, enabling programs to leverage local resources and affiliations. The different resources and strategies used to develop street medicine programs has led to a variety of program operations that fall under the street medicine model. Although each program operates mostly independently of other street medicine programs, there are certain features that are uniform across programs such as their philosophies and values as well as the challenges they encounter. Variable components such as the program model, scope of practice, and affiliations are influenced by the local context in which the street medicine operates. These variable components are essential to the flexible nature of the street medicine model that allows each program to leverage local resources and respond to the unique needs of those in their local or regional area. Street medicine programs often serve as a bridge between formal health entities and people on the streets who are usually disengaged from these formal health networks. Programs’ use of low-barrier and harm-reduction health interventions are key features that facilitate the success of street medicine across the U.S. As programs across the U.S. continue to emerge and develop, future research should consider robust and sustainable funding options for street medicine. This work is essential to ensuring that street medicine programs are financially viable and able to continue their work to meet the needs of PEH. In addition to investigating how to design programs for sustainability, a stronger emphasis on measuring the impact of street medicine programs on health outcomes is important. Programs should also consider research that investigates how the inclusion of people with lived experience and harm-reduction principles can be integrated with street medicine teams.

## Figures and Tables

**Table 1:** Street medicine program models and corresponding features.

Type of Street Medicine Program Model	Staffing Requirements	Overview
**Staff Model**	At least one paid, licensed medical provider. AND At least one paid staff dedicated to social services, logistical, operational, or administrative work (e.g., caseworker, community health worker, management staff).	Staff models are not dependent on volunteers to maintain program operations or provide healthcare services. Volunteers within this model perform roles that support the program’s efforts.
**Staff-Volunteer Hybrid Model**	At least one paid, licensed medical provider. OR At least one paid staff dedicated to social services, logistical, operational, or administrative work (e.g., caseworker, community health worker, management staff).	Hybrid models have at least one paid staff member plus volunteers dedicated to a role essential for program operations.
**Volunteer Model**	No paid personnel At least one volunteer dedicated to a role of management staff. Partnerships with licensed medical providers that volunteer consistently with the program	Volunteer models are completely dependent on volunteers to maintain program operations and provide healthcare services.

**Table 2:** Summary of participating street medicine programs.

Street Medicine Program	Level of Care	Affiliation	Program Model
Program A	3	FQHC	Staff
Program B	3	FQHC	Staff
Program C	3	FQHC	Staff
Program D	3	FQHC	Staff
Program E	3	Academic Institution	Staff
Program F	2	Academic Institution	Volunteer
Program G	2	County Entity	Staff
Program H	2	Nonprofit	Hybrid
Program I	2	County Entity	Staff
Program J	2	Nonprofit	Staff
Program K	2	FQHC and Academic Institution	Staff
Program L	1	Academic Institution	Volunteer
Program M	1	Academic Institution	Volunteer

## Data Availability

The datasets presented in this article are not readily available because participants were told that their data would be used in aggregate during the consent process.

## Appendix A. Interview Guide for Street Medicine Study

My name is Teresa Medellin, and I am a fourth-year student at the University of Texas in Austin. I’m conducting a series of interviews as part of my senior thesis. The goal of my senior thesis is to characterize the current nature and scope of street medicine programs in the United States. I have investigated many street medicine programs, and I have recognized that street medicine programs have rarely been studied collectively. Thus, my thesis will document the features of multiple programs and develop a conceptual scheme of the characteristics of street medicine programs. The purpose of the interviews is to gain insight into the specific facilitators and barriers of each program as well as learn about each program’s development, operations, philosophy, and vision for the future. I will be interviewing social service and healthcare providers from street medicine programs from a broad geographic spread in the United States, and I will be interviewing different program types (e.g., community health centers, hospital-based programs, and public entities).

This interview will take 30–60 min, and my questions relate only to your professional experience as a [insert profession] on the [X street medicine team]. I won’t ask any questions that are not pertinent to your work duties and responsibilities. Your decision to speak with me is voluntary. You can also refuse to participate or answer any questions, and you may stop this interview at any time. If, at a later time, you’d like to retract certain statements, you may do so. Your information will be stored on a password-protected computer. What you share with me during the interview will be used with the information we gather from the other people I am interviewing.

My senior thesis will have no direct benefit to you. However, it will allow for an in-depth characterization of existing street medicine programs to inform the street medicine community, healthcare systems, and funders about street medicine programs. If you agree to participate, I’d like to record our interview so that I can capture all of the important information that you share with me. Anything you say is on the record, but if there’s something you want to tell me off the record, let me know, and I’ll pause the recording. Also, if there’s something you’ve said on the record you want taken off, let me know at any time during or after the interview, and I can remove it.

Now, I have three questions for you. The first is, are you willing to participate in this interview? Do you give me permission to record this interview? In addition, do you give me permission to contact you at a later time if I have additional questions?

If you have questions about this project, you may contact me at this email address (tmedellin@utexas.edu) or the University of Texas institutional review board (512-232-1543 or irb@austin.utexas.edu).

Interview Questions:

1. What is your role on the street medicine team? How long have you been in this role?
2. What is the philosophy and ethos of your program? Does your program have any stated values?
3. Describe your program.
  Follow up questions to ask if not addressed during description of program:
  - How did the program develop?
  - Describe the program’s resources and funding.
  - What is the team composition?
  - Does the program use an EHR?
  - What are the demographics of your patient population?
4. How does the program define patient success and program success?
5. What factors facilitate the effectiveness of your program?
6. What challenges does the program face?
7. Much of the literature describing barriers to care for the unsheltered homeless population documents mistrust of healthcare workers as a significant barrier. How does your program address this barrier?
8. How does the team identify which communities to visit?
9. What is the frequency with which the team visits communities?
10. What services does your program offer?
  - Are there some services that are more frequently requested or needed?
11. Do the health recommendations offered by care providers on the street differ from the health recommendations that are generally given in a clinical setting to housed patients? If yes, describe how they differ.
12. What outcomes does your program track?
13. What guidelines does your program have for quality management?
14. How has the pandemic impacted the program?

Note: The following probing questions will be used, as appropriate, throughout the interview:

Would you give me an example?

Can you elaborate on that idea?

Would you explain that further?

I’m not sure I understand what you’re saying.

Is there anything else?
